# EBRNetwork – a call to action for evidence-based research

**DOI:** 10.1186/1745-6215-16-S3-P10

**Published:** 2015-11-24

**Authors:** Karen A Robinson, Klara Brunnhuber, Robin Christensen, Peg Ford, Maureen Dobbins, Bertil F Dorch, Marlies Leenars, Hans Lund, Malcolm Macleod, Mona Nasser, Hanna Nykvist, Matt Westmore

**Affiliations:** 1 Johns Hopkins University School of Medicine, Baltimore, Maryland, USA; 2 Clinical Evidence and Best Practice at BMJ Publishing Group, London, UK; 3 Odense University Hospital, the Parker Institute, University of Southern Denmark, Odense, Denmark; 4 Ovarian Cancer Alliance of San Diego, Coronado, CA, USA; 5 School of Nursing at McMaster University, Ontario, Canada; 6 University of Southern Denmark, Funen, Denmark; 7 SYstematic Review Centre for Laboratory animal Experimentation (SYRCLE), Radboud University Medical Center, Nijmegen, The Netherlands; 8 University of Southern Denmark & Bergen University College, Odense M, Denmark; 9 Centre for Clinical Brain Sciences at the University of Edinburgh, Edinburgh, UK; 10 Plymouth University Peninsula School of Medicine and Dentistry, Plymouth, UK; 11 Bergen University College, Bergen, Norway; 12 NIHR Evaluation, Trials and Studies Coordinating Centre, University of Southampton, Southampton, UK

## Background

Use of earlier research is needed to provide the rationale for starting a study and a context in which to set the study results. Explicit use of earlier research, through the conduct of a systematic review, is also necessary for the design of an efficient and informative study. Decisions about design informed by systematic review include the selection of meaningful outcomes assessed in a way that enables synthesis across studies; this is especially needed in areas where core outcome sets do not yet exist. Yet research shows that there is inadequate and biased consideration of earlier research.

## Methods

In Bergen, Norway, in December 2014, the Evidence-Based Research Network (EBRNetwork) was initiated to promote the efficient and explicit use of existing research when new research is planned.

## Results

We will present the aims, structure and activities of the EBRNetwork. Current activities include using peer-reviewed publications and social media to inform and engage researchers, funders, editors and the public. We will present for consideration a preliminary version of the Bergen Statement on Evidence-Based Research.

## Conclusion

The design of new studies, including the choice of outcomes, should be informed by prior research. The new EBRNetwork is an international collaboration that aims to ensure that no new studies are approved, funded or published without systematic review of existing evidence; and works towards more efficient production, updating and dissemination of systematic reviews. The Network issues a call for participation.

